# Bearing Fault Diagnosis Based on Time–Frequency Dual Domains and Feature Fusion of ResNet-CACNN-BiGRU-SDPA

**DOI:** 10.3390/s25133871

**Published:** 2025-06-21

**Authors:** Jarula Yasenjiang, Yingjun Zhao, Yang Xiao, Hebo Hao, Zhichao Gong, Shuaihua Han

**Affiliations:** College of Intelligent Manufacturing and Industrial Modernization, Xinjiang University, Urumqi 830017, China; 107552404325@stu.xju.edu.cn (Y.Z.); 107552204305@stu.xju.edu.cn (Y.X.); haohebo@stu.xju.edu.cn (H.H.); gongzhichao@stu.xju.edu.cn (Z.G.); 18238762680@163.com (S.H.)

**Keywords:** fault diagnosis, time–frequency dual domain, residual network, bidirectional gated recurrent network

## Abstract

As the most basic mechanical components, bearing troubleshooting is essential to ensure the safe and reliable operation of rotating machinery. Bearing fault diagnosis is challenging due to the scarcity of bearing fault diagnosis samples and the susceptibility of fault signals to external noise. To address these issues, a ResNet-CACNN-BiGRU-SDPA bearing fault diagnosis method based on time–frequency bi-domain and feature fusion is proposed. First, the model takes the augmented time-domain signals as inputs and reconstructs them into frequency-domain signals using FFT, which gives the signals a bi-directional time–frequency domain receptive field. Second, the long sequence time-domain signal is processed by a ResNet residual block structure, and a CACNN method is proposed to realize local feature extraction of the frequency-domain signal. Then, the extracted time–frequency domain long sequence features are fed into a two-layer BiGRU for bidirectional deep global feature mining. Finally, the long-range feature dependencies are dynamically captured by SDPA, while the global dual-domain features are spliced and passed into Softmax to obtain the model output. In order to verify the model performance, experiments were carried out on the CWRU and JNU bearing datasets, and the results showed that the method had high accuracy under both small sample size and noise perturbation conditions, which verified the model’s good fault-feature-learning capability and noise immunity performance.

## 1. Introduction

With the continuous development of science and technology, the intelligent digitalization of the machinery industry is proceeding at a rapid pace [1]. Utilizing cutting-edge computer technology, intelligent diagnosis of traditional mechanical parts has also gained popularity [2,3,4]. The reliable operation of wind turbines, high-speed trains, new-energy vehicles, and advanced machinery in various industries depends on bearings, which are an important part of mechanical gears [5,6,7].

Due to the intricacy of the bearings’ operating environment, bearing load, and monitoring challenges, bearings are very vulnerable to a range of operational failures, which increases the risks to employee safety and security, as well as to corporate production. Bearing failures account for 45% to 55% of mechanical equipment failures [8], and they account for 40% of motor failures [9]. It is crucial to perform fault diagnosis and safety assessment of bearings in order to ensure their safe operation, prevent significant production accidents, and prevent personnel injuries resulting from bearing failures [10]. With its high accuracy rate and a potent feature extraction capability that surpasses traditional machine learning, Deep Learning, a state-of-the-art fault diagnosis technology, offers a completely new perspective and methodology for fault diagnosis. It is based on big data technology and increasing computer arithmetic power. Convolutional Neural Networks (CNNs) [11,12,13], Residual Networks (ResNet) [14], and Gated Recurrent Unit Models (GRU) [15,16], for example, have yielded satisfactory results when processing long sequence signals in the field of bearing fault diagnosis.

To solve the problems of low detection accuracy and recognition difficulties for weak early fault signals of rolling bearings, Shan et al. [17] proposed an approach for weak early fault signal detection for bearings that roll based on a double-coupling Duffing system and VMD. The foundation for the determination was provided, and the impact of the system’s initial value on the response characteristics of the dual-coupled Duffing system was examined. With the lowest signal-to-noise ratio, the suggested technique could identify the weakest fault signal. Repeated information interference and complicated nonlinear and multiscale features are necessary to handle rolling bearing noise in rotating machinery. Li et al. [18] suggested an improved deep residual shrinkage network (eDRSN) and multi-scale channel hybrid convolutional network (MSCMN) to increase the accuracy of fault identification and feature learning in industrial settings. In order to offer comprehensive information, the MSCMN applied to the first and intermediate network layers captured multi-scale features from vibration signals. Conventional linear layer representations are typically insufficient because industrial vibration signals are extremely noisy and nonlinear. Sulaiman et al. [19] proposed an eDRSN with a Kolmogorov–Arnold Network Linear Layer (KANLinear), which combines linear transformations with B-spline interpolation, to capture both linear and nonlinear information for better threshold learning. The model exhibited enhanced robustness and diagnostic accuracy in noisy real-world scenarios. A hybrid deep learning model called MSRN-EGRU was introduced by Liao et al. [20] with the objective of addressing CNNs’ shortcomings in multi-scale feature extraction and temporal feature capture. In order to enhance the model’s feature representation and effectively extract local features, an MSRN was created by incorporating residual connectivity and multi-scale structure into CNNs. In order to further extract temporal data, the extracted local features were then input into an EGRU. The EGRU enhances the GRU structure and incorporates scaling exponential linear units (SELUs), which improves the memory and nonlinear modeling capabilities.

The ability of the above model for bearing fault characterization has been improved, but due to the long life of bearings in general, and the difficulty of fault data collection, bearing fault diagnosis work often presents the problem of scarcity of fault samples [21,22], which causes the model’s accuracy to decrease. How to accurately mine effective features in the case of small sample sizes is also an important research direction in the field of bearing fault diagnosis [23]. A model was encouraged to make the most of few labeled data with a novel loss function based on positive and negative losses. To make use of class information from labeled data, Cai et al. [24] created a ternary channel graph building method and used label propagation. In order to address the issues of non-stationary fault signals, which make feature extraction challenging, and small sample sizes, which results in low fault diagnosis accuracy, Zhai et al. [25] proposed a novel rolling bearing fault diagnosis technique based on a synchronized compressed wavelet transform (SWT) and transfer residual convolutional neural network (TRCNN). Li et al. [26] suggested memory-enhanced prototype meta-learning (MAPML) for small-sample defect identification. In order to improve the model’s capacity to correctly categorize zones, a dynamic prototype tuning module was suggested. This module uses a memory enhancement approach to dynamically and adaptively improve class prototypes. Fan et al. [27] proposed a new lightweight DDPM that integrates multi-dconv head transposition attention (MHTA) into the U-Net architecture, which significantly reduces computational complexity by combining point-by-point and deep convolution to shift the attention computation from the pixel dimension to the channel dimension. The accuracy of fault diagnosis was improved even under limited sample conditions.

In summary, the current model primarily uses a single optimized data processing method to increase sample richness under small sample sizes, which may improve the model training effect. However, it is vulnerable to risks like gradient explosion and overfitting, and its generalization and robustness may be inadequate in real-world engineering application scenarios. Furthermore, research has indicated that under specific complex working situations, bearing fault characteristics can greatly change. For instance, the accuracy of a model may be significantly reduced if it only uses one time-series dataset and ignores the deeper correlation of problems in the frequency domain, as in the diagnostic task under noise-disturbed conditions.

To address the challenges mentioned above, this paper proposes a ResNet-CACNN-BiGRU-SDPA bearing fault diagnosis method based on time–frequency dual domains and feature fusion. The method explores key time–frequency domain information and fuses long sequences of global features through dual channels in order to learn rich fault detail features in samples. The main contributions and novelties of this paper can be summarized as follows:

- A multi-window overlapping data enhancement and sampling technique is proposed to deal with sparse fault samples and provide rich fault sample support for subsequent diagnosis. Then, the Cooley–Tukey algorithm is introduced to convert the time-domain signals used in traditional fault diagnosis to the frequency domain, highlighting the periodic local features of the signals, which greatly expands the sensory field of the signals for multi-dimensional features, while simplifying signal representations, enabling the subsequent model to mine richer and more comprehensively detailed features.
- A CACNN structure is proposed to decompose the features and calculate the weight fusion of the input features in both the horizontal and vertical directions. This structure eliminates the problems of incomplete local feature extraction and overfitting when extracting long sequence features in the frequency domain with minimal computation, while preventing the model from falling into local optima and feature loss. In addition, while the CACNN extracts the local frequency-domain features, another channel uses the ResNet structure to process the long sequence time-domain features. This maximizes the extraction of the detailed features of the model in both the time and frequency domains for the dual channels, and improves the performance of the model’s feature extraction.
- The two-layer BiGRU structure helps explore and extract features by looking at both time and frequency, allowing it to understand long-distance relationships between local features and rebuild the overall signal, while gathering detailed bi-directional features. The SDPA structure dynamically captures complex feature information and adapts to changing noise conditions, which enhances the adaptability and robustness of the intelligent diagnostic model to noise, forms a complete and efficient diagnostic system, and expands the scope of the intelligent diagnostic model and its reliability in practical engineering applications.

The remaining sections of this paper introduce three problems, and Section 2 describes the role and application principles of each underlying module of the ResNet-CACNN-BiGRU-SDPA method. Section 3 describes the improvement method used in this paper to address the above issues and the structure of the modules, and describes the diagnostic flow of the model and the model parameter settings. Section 4 describes the experimental data sources and experimental flow design, and details the small-sample experiment, noise perturbation experiment, ablation experiment, T-SNE visualization experiment, and confusion matrix experiment used to verify the model performance and reliability from different perspectives.

## 2. Related Work

In this section, we present related work that clarifies the central role and applicable location of the CNN, BiGRU, and SDPA structures used in this paper.

### 2.1. CNN

CNNs’ exceptional high-dimensional case performance, feature extraction, and local perception have led to their widespread application in recent years. A CNN’s main layers are convolutional and pooling layers [28]. The convolutional layer’s role involves feature extraction, whereby local features are extracted from the source signal via convolutional processes. Assuming that the data input is *X*, the convolution kernel is *W*, the bias term is *b*, and the output result of the convolutional operation is *Y*, and the operational method of the convolutional layer can be explained as below.

(1) Y=σ(W∗X+b)

The convolutional operation is represented by ∗ in Equation (1), the convolutional layer’s activation function is σ. The time-series signals of the bearings examined in the paper are one-dimensional features, and a CNN performs well in extracting spatial data from the input signals. However, a CNN is not very good at capturing the dependency relationships of the long time sequences, and it is prone to issues like inadequate processing of time series signals, poor model interpretability, and overfitting when processing one-dimensional time-series signals [29].

### 2.2. Bi-Directional Gated Circulation Unit

The Gated Recurrent Unit (GRU) is a solution to the gradient vanishing issue in Recurrent Neural Networks (RNNs) when working with long sequence signals [30]. This unit is ideal for handling long sequence signals because it incorporates a gating mechanism to better regulate the information flow.

A GRU’s basic idea is to use two gating mechanisms—an update gate and a reset gate—to control the updating and resetting of the concealed state. The following equation shows the reset gate:

(2) $$r_{t}=\sigma \left(W_{r}\cdot \left[h_{t-1},x_{t}\right]\right)$$

When using Equation (2), the reset gate’s output is $r_{t}$, its weight matrix is $W_{r}$, its hidden state from the previous moment is $h_{t-1}$, its input is $x_{t}$, and its sigmoid activation function is σ. The reset gate regulates the amount of influence the hidden state has on ${\tilde{h}}_{t-1}$ from the previous moment. The output of the reset gate $r_{t}$ is obtained by removing the influence of the hidden state $h_{t-1}$ of the previous instant, assuming that $h_{t-1}$ has no link to the current hidden state.

The input $x_{t}$ at the present time and the hidden state $h_{t-1}$ at the past time are both calculated by the update gate:

(3) $$z_{t}=\sigma \left(W_{z}\cdot \left[h_{t-1},x_{t}\right]\right)$$

In Equation (3), $z_{t}$ is the output of the update gate and $W_{z}$ is the weight matrix of the update gate;

Candidate hidden state computation:

(4) $${\tilde{h}}_{t}=\tanh\left(W\cdot \left[r_{t}⊙h_{t-1},x_{t}\right]\right)$$

In Equation (4), ${\tilde{h}}_{t}$ is the candidate hidden state, *W* is the weight matrix, and ⊙ denotes element-by-element multiplication;

The current state hidden state is calculated as follows:

(5) $$h_{t}=\left(1-z_{t}\right)⊙h_{t-1}+z_{t}⊙{\tilde{h}}_{t}$$

In Equation (5), $h_{t}$ is the hidden state at the current moment, and the current moment hidden state is obtained according to the calculation of Equations (5) and (6). The multiplication operation at the element level is represented by the equation above. To better manage long sequence data, the GRU can determine, to a certain extent, which information should be updated and which should be forgotten using the update gate and reset gate processes.

**Figure 1:**
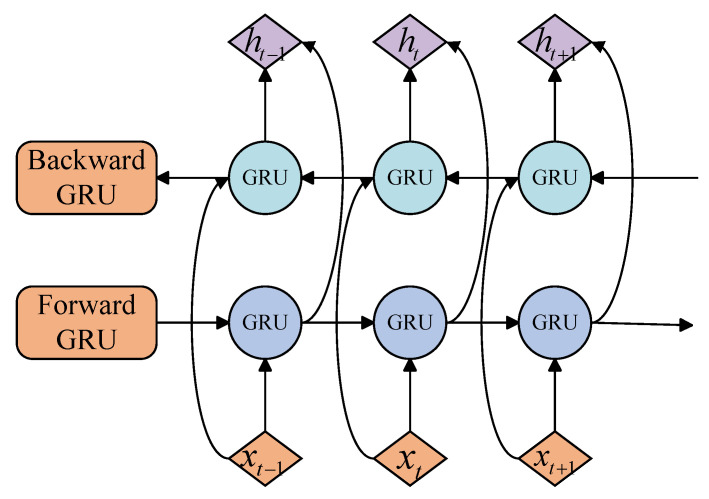
BiGRU structure.

As seen in Figure 1 of the BiGRU structure, a BiGRU is a model that is based on a GRU and incorporates forward and backward feature considerations for sequence information [31]. It is composed of two separate GRUs. Its forward and backward GRUs are represented in the following equation:

(6) $$\vec{h_{t}}=\mathrm{GRU}\left(x_{t},\vec{h_{t-1}}\right)$$

(7) $$\overset{\leftarrow }{h_{t}}=\mathrm{GRU}\left(x_{t},\overset{\leftarrow }{h_{t+1}}\right)$$

In Equations (6) and (7), $\vec{h_{t}}$ and $\overset{\leftarrow }{h_{t}}$ are the forward and backward GRU hidden states; $x_{t}$ is the input at the current moment; $\vec{h_{t-1}}$ and $\overset{\leftarrow }{h_{t+1}}$ are the bi-directional hidden states of the previous moment and the next moment; and the bi-directional GRU processes the inputs sequentially from the beginning to the end of the sequence, captures the forward and reverse feature signals, and splices them together to obtain the final output $h_{t}$ shown in Equation (8).

(8) $$h_{t}=\left[\vec{h_{t}};\overset{\leftarrow }{h_{t}}\right]$$

### 2.3. Scaling Dot Product Attention Mechanism

**Figure 2:**
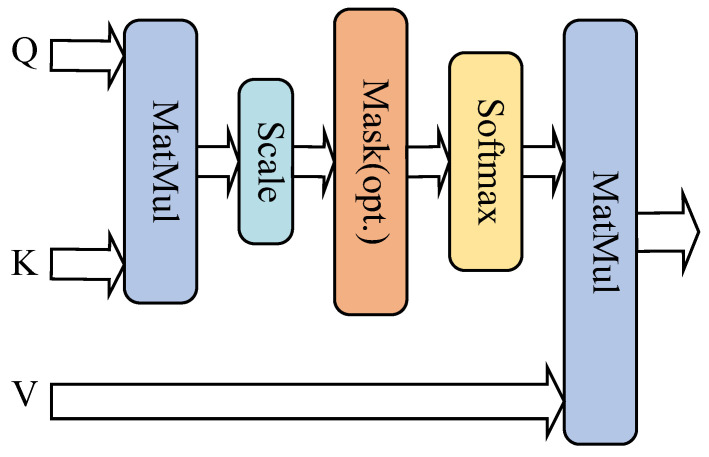
SDPA structure.

One of the main elements of the Transformer model is the scaled dot product attention mechanism (SDPA). The ability of the SDPA to process all of the sequence’s elements at once through matrix operations makes it more computationally efficient than traditional attention mechanism algorithms. It also has a strong ability to capture long-distance dependencies, which is particularly crucial when handling bearing failure signals with complex structures and long-distance dependencies [32]. As seen in Figure 2, the structure of the SDPA and its formula can be expressed as follows: It calculates the dot product between the query vector (Query), the key vector (Key), and the value vector (Value) in order to measure the correlation. It also stabilizes the computational process by scaling operations.

(9) $$\mathrm{Attention}\left(Q,K,V\right)=\mathrm{softmax}\left(\frac{QK^{T}}{\sqrt{d_{k}}}\right)V$$

In Equation (9), *Q* is the query matrix, *K* is the key matrix, *V* is the value matrix, and $d_{k}$ is the dimension of the vectors. The cosine similarity between the *Q* query matrix and *K* is used to calculate the degree of correlation between the two vectors, and dividing by $\sqrt{d_{k}}$ prevents the issues of gradient exploding or vanishing because of the excessively large dot product in the high-dimensional space. Furthermore, Softmax determines the attention weights, which are then applied to the input signal to improve the characterization of long sequences.

## 3. Proposed Method

In this section, we first introduce the ResNet-CACNN-BiGRU-SDPA method and diagnostic logic proposed in this paper and then explain the contributions of each part in detail. The method in this paper constructs a bi-domain feature association model, which focuses on solving the problem of incomplete and insufficient feature extraction in general methods, and enhances the model’s adaptability to noisy environments through the SDPA structure.

### 3.1. ResNet-CACNN-BiGRU-SDPA Modeling

**Figure 3:**
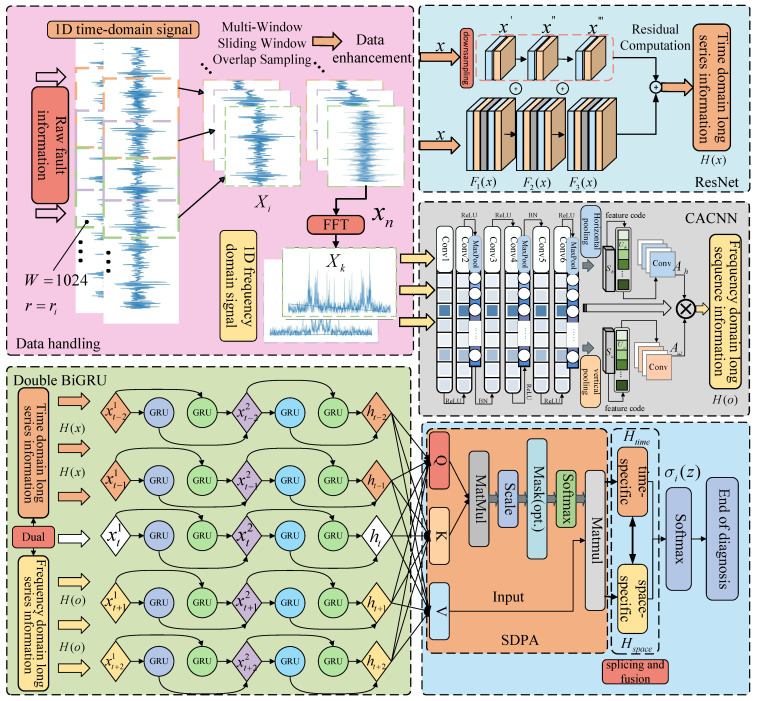
ResNet-CACNN-BiGRU-SDPA model structure.

General bearing fault diagnosis methods only focus on the time series signal of bearing faults or increase the number of training samples through different data processing methods, but they ignore the frequency-domain fault information, and the fault signal extraction is not comprehensive. Moreover, traditional models generally only have a good diagnostic effect when there are more training samples or the fault signal is smooth, but when there is a lack of fault samples or noise perturbation, models are prone to overfitting and a sharp decline in classification accuracy; therefore, a ResNet-CACNN-BiGRU-SDPA network model based on the time–frequency bi-domain and feature fusion is proposed, as shown in Figure 3.

The model, which includes a ResNet, FFT, CACNN module, two-layer BiGRU module, and SDPA attention mechanism, takes the bearing’s original time-sequence signal as input. It then uses FFT to reconstruct the one-dimensional time-domain signal to the frequency domain, extracts the time–frequency domain long-sequence hybrid features based on the ResNet and CACNN structure, uses the SDPA to capture the feature long-distance dependencies, and feeds the data into the classifier. The following are the precise steps involved in putting this strategy into practice:

1. One-dimensional time-domain signals are converted into one-dimensional frequency-domain signals using the Cooley–Tukey technique, based on a multi-window sliding-window overlapping sampling technique to gather signals from the original data, which enriches the acquired data features, and then adding Gaussian white noise to improve the model’s adaptability to the noise;
2. With the goal of conducting local feature exploration in the time-domain direction, the acquired one-dimensional time-domain signals are fed into the ResNet network. Meanwhile, the CACNN module is used to conduct local feature exploration in the frequency-domain direction, meaning that sensitive fault time–frequency-domain information, H(x) and H(o), is mined by the two-way mining of ResNet and CACNN;
3. The temporal and spatial BiGRU(T-S BiGRU) module receives the signal from feature mining, and the forward and reverse information is used to investigate the deeper global features in the time–frequency domain and to capture long dependencies in the sequence data;
4. The time–frequency features extracted from the T-S BiGRU layer are used to measure the correlation with the dot product operation between the query vector (Query), the key vector (Key), and the value vector (Value) in the SDPA mechanism, and the outputs of the temporal and spatial features $H_{time}\in R$ and $H_{space}\in R$ after the dot product operation;
5. Splicing and fusing the above spatio-temporal features and obtaining the model probability distribution $\sigma_{i}\left(z\right)$ with Softmax completes the fault classification task, and the following is the Softmax mathematical expression:
  (10) $$\sigma_{i}\left(z\right)=\frac{e^{z_{i}}}{\sum_{j=1}^{n}e^{z_{j}}}$$
  In Equation (10), *e* is a natural constant and $z_{i}$ is the *i*th element of the input vector.

The following Section 3.2, Section 3.3 and Section 3.4 describe in detail the establishment of the time–frequency dual-channel domain ResNet and CACNN structure, elaborating on their necessity for solving the aforementioned problems, such as the difficulty of feature extraction in the case of small sample sizes and noise perturbations.

### 3.2. FFT Conversion of One-Dimensional Bearing Signals

The fundamental idea behind FFT is to discretize the computation of the DFT into smaller blocks in order to speed up and reduce the computation. The DFT and its opposite can be calculated rapidly with the FFT algorithm [33]. The defining equation for the DFT is

(11) $$X_{k}=\sum_{n=0}^{N-1}x_{n}\cdot e^{-i2\pi kn/N}$$

In Equation (11), $X_{k}$ is the *k*th sample in the frequency domain, $x_{n}$ is the *n*th sample in the time domain, *N* is the number of samples, and *i* is the imaginary unit.

The FFT decomposes the DFT computation into smaller sub-problems through the Divide and Conquer strategy, the Cooley–Tukey algorithm used in this paper is as follows:

(12) $$X_{k}=\sum_{n=0}^{N/2-1}x_{2n}\cdot e^{-i2\pi kn/(N/2)}+e^{-i2\pi k/N}\sum_{n=0}^{N/2-1}x_{2n+1}\cdot e^{-i2\pi kn/(N/2)}$$

In Equation (12), the summation term 1 is the FFT of the even-indexed subsequence, the summation term 2 is the FFT of the odd-indexed subsequence, and $e^{-i2\pi k/N}$ is the Twiddle Factor. By decomposing the input sequence $x_{n}$ into even-indexed and odd-indexed subsequences, applying the FFT recursively to the subsequence and merging the results, the final FFT transform result $X_{k}$ is obtained as shown in Equation (13). After converting the time-domain signal to a frequency-domain signal, the fault characteristic frequency is clearer and the signal energy distribution is also displayed, which has an obvious improving effect on fault characteristics under noise and other complex working conditions.

**Figure 4:**
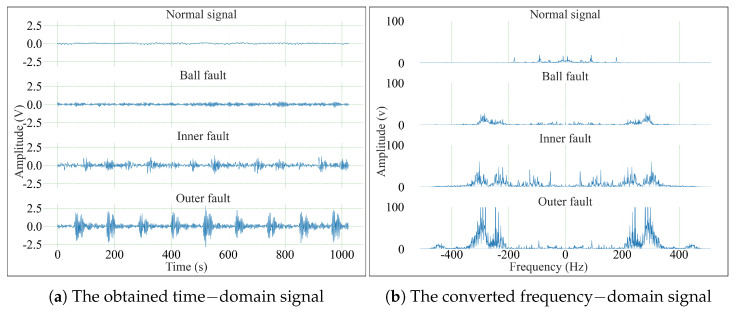
FFT example.

As an example, this paper uses the CWRU dataset 007 type bearing normal baseline signals, rolling element, and inner and outer ring fault signals (Figure 4a for the time-domain signals, and Figure 4b for the frequency-domain signals converted by the Cooley–Tukey algorithm). Since FFT only makes sense for positive frequencies, and we focus on the positive frequency components of the frequency-domain signals. The long-sequence timing features are converted to the frequency domain and then fed into the subsequent CACNN module. The CACNN module learns the frequency components and energy distribution features of the signals in the positive frequency domain, thereby extracting multidimensional information from the fault-related directional data.

### 3.3. ResNet Network and Residual Blocks

The inclusion of Residual Learning and Shortcut Connections, two technologies that enable the network to train very deep models more effectively, is the fundamental innovation of ResNet [34] for addressing the issue of vanishing gradients in deep networks and to train extremely deep models more effectively.

ResNet’s fundamental component, the residual block, addresses the problem of gradient vanishing in deep network environments by introducing a kind of ‘leapfrog connection’ that allows the network to learn the residual mapping between inputs and outputs instead of the mapping itself [35]. This facilitates model training in extremely deep networks. If the input is *x* and the output is $H\left(x\right)$, the residual block can be described as follows:

(13) H(x)=F(x)+x

**Figure 5:**
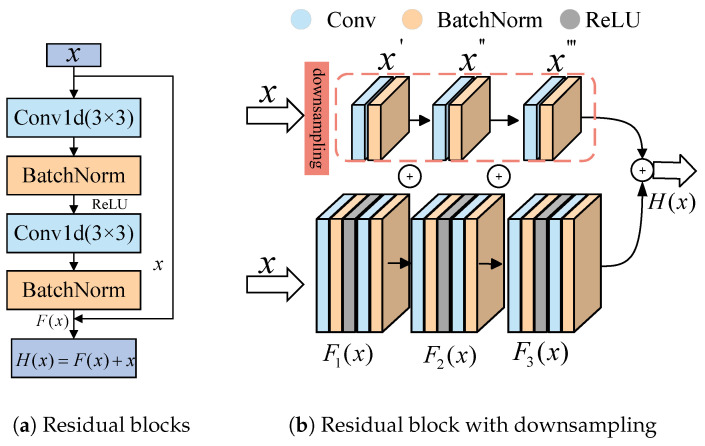
Two kinds of residual blocks.

The structure of the residual block in Figure 5a illustrates how $F\left(x\right)$ in Equation (13) may be viewed as the fusion result of the convolutional layer, batch normalization, and activation function ReLU operations in the residual block, the input feature *x* first passes through the 3 × 3 convolutional layer, and then accelerates the training process and activates the feature by BatchNorm1d and ReLU, and finally passes through a 3 × 3 convolutional layer, as well as BatchNorm1d, to obtain the output $F\left(x\right)$, and the final output $H\left(x\right)$ is obtained by summing the input *x* with the output of the convolutional layer $F\left(x\right)$.

In the residual block, the dimensions of the input and output may sometimes not match; at this time, the downsampling method is introduced to adjust the input dimension to match the output dimension [36], as shown in Figure 5b. The input *x* is obtained through a 1 × 1 convolutional layer and a batch normalization layer to obtain $x^{*}$; at this time, the output of the residual block is H(x).

(14) $$H\left(x\right)=F\left(x\right)+x^{*}$$

### 3.4. CACNN Model

**Figure 6:**
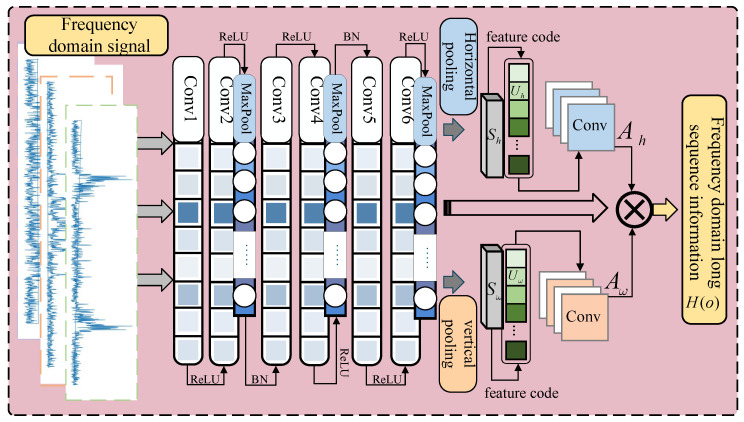
CACNN model.

By introducing the CoordAttention fusion convolution structure, this paper proposes a CACNN frequency-domain feature extraction method, as illustrated in Figure 6, to address the issues of incomplete feature extraction and overfitting in conventional CNN models when working with longer time series features.

The characteristics in this study are compressed and encoded by pooling in the vertical as well as horizontal directions using the conventional convolutional pooling procedure, in order to produce a complementary effect. Additionally, the feature encoding operation converts the bi-directional signals, both horizontal and vertical, into a more expressive and discriminative form. Convolution is then used for local feature extraction in the frequency domain to further improve the feature-encoded features and incorporate them into weights.

The proposed CACNN model has a simple structure, but can significantly improve the feature learning performance without additional computational effort, and has a better feature mining effect in the feature perception of non-smooth signals, such as noise disturbances, to enhance its fault feature learning capability.

For the purpose of extracting the local characteristics of the source data, a convolutional structure including numerous convolutional layers, a layer for batch normalization, and an activation function, initially undergoes initial feature learning on the fault signal. The channel attention is broken down into two concurrent one-dimensional feature encodings to provide the integrated spatial coordinate information. Assuming that the frequency-domain input is $H_{i}\in R^{C^{\prime }\times H^{\prime }\times W^{\prime }}$, the global pooling operation is performed in the horizontal and vertical directions, respectively, to obtain the global horizontal and vertical feature vectors $S_{h}$ and $S_{\omega }$, as shown in Equations (15) and (16):

(15) $$S_{h}=\frac{1}{H^{\prime }}\sum_{i=1}^{H^{\prime }}H_{i}\left[:,i,:\right]$$

(16) $$S_{\omega }=\frac{1}{W^{\prime }}\sum_{j=1}^{W^{\prime }}H_{i}\left[:,:,j\right]$$

The output of the above equation is feature coded in the horizontal and vertical directions to generate the bidirectional attentional search domains $U_{h}$ and $U_{\omega }$, as shown in Equations (17) and (18):

(17) $$U_{h}=\sigma \left(W_{uh}\cdot S_{h}+b_{uh}\right)$$

(18) $$U_{\omega }=\sigma \left(W_{u\omega }\cdot S_{\omega }+b_{u\omega }\right)$$

The bi-directional attentional search domain is transformed by convolution into bi-directional attentional weights $A_{h}$ and $A_{\omega }$, as shown in Equations (19) and (20):

(19) $$A_{h}=\mathrm{Conv}\left(U_{h}\right)$$

(20) $$A_{\omega }=\mathrm{Conv}\left(U_{\omega }\right)$$

The generated horizontal and vertical attention weights are subjected to an element-by-element multiplication operation with the original features $H_{i}$, as indicated in Equation (21), and the aim is to produce the model output H(o) by improving the significant characteristics and suppressing the unimportant ones:

(21) $$H\left(o\right)=H_{i}⊙\left(A_{h}\cdot A_{\omega }\right)$$

If one wants to mine the best signal output in the frequency-domain state, the proposed CACNN model can detect multidimensional information in the signals and perform deep feature sensing of the multidimensional information for bearing frequency-domain fault signals.

### 3.5. Model Parameters

The ResNet-CACNN-BiGRU-SDPA model network parameters are shown in Table 1. The raw faulty input signal dimension is 1024 × 10, and each sample has 1024 time steps and 10 channels. The CACNN layer contains six one-dimensional convolutional layers and three maximum pooling layers, and the spatial sequence features are extracted and compressed by the convolutional operation and maximum pooling. The input features are gradually upgraded to 64 × 64, while the spatial location information of the features is enhanced to obtain the 64 × 64 frequency-domain feature output, while for the time-domain direction, the enhanced time-domain signal is input into three ResidualBlock1 residual blocks. Each residual block consists of two layers of Conv1d convolution and downsampling operations, and the final output is a 1024 × 128 dimensional feature, with the time-domain and frequency-domain features extracted through the spatio-temporal BiGRU layer. The BiGRU layer includes a two-layer Time–BiGRU layer and a two-layer Space–BiGRU layer, and the output dimensions are 1024 × 128 and 64 × 128. In order to highlight the important time step and feature information, the spatio-temporal features after BiGRU processing are feature-weighted through the SDPA, and the spatio-temporal features are spliced and fused to obtain the output with 256 dimensions, and finally mapped to the final category labels through the fully connected layer to complete the model classification task.

### 3.6. Troubleshooting Process

**Figure 7:**
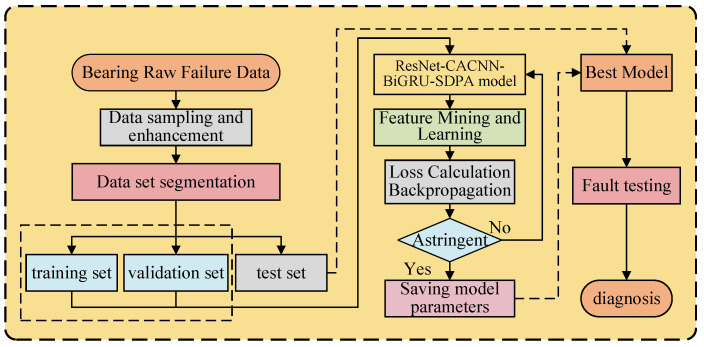
Fault diagnosis process.

The fault diagnosis flow of the ResNet-CACNN-BiGRU-SDPA method based on time–frequency dual domains and feature fusion is shown in Figure 7, which is divided into three main stages:

1. Preparation of data. The original bearing fault signal is subjected to multi-window sliding window overlapping sampling and Gaussian noise data enhancement, and the training, validation, and test sets are separated in a 7:2:1 ratio;
2. Feature extraction and model training. In this work, we used the Adam optimizer, with the learning rate at 0.0003, the number of training rounds at 50, and the model batch size at 32. The model is then given data from the processed training and validation sets, and the training and validation process loss is computed. After the model converges, the optimal model parameters are saved until the end of the iterative process with the designated number of rounds. The model parameters are then updated using backward gradient propagation. The cross-entropy function shown below is used to calculate the training loss:
  (22) $$L=-\sum_{i=1}^{n}y_{i}\log{\hat{y}}_{i}$$
  In Equation (22), *n* is the number of categories, $y_{i}$ is the one-hot encoding of the true label, and ${\hat{y}}_{i}$ is the probability predicted by the model, which maximizes the probability of the correct category, while minimizing the predicted-true distribution difference.
3. Model classification test. We tested the best model given above on the test set, evaluated the model’s performance, and tested the accuracy and fault classification effect of a model that was trained on new data.

## 4. Experimental Verification

In this section, for the purpose of verifying the feature extraction ability and anti-noise-influence performance of the suggested method in small sample conditions, we selected the CWRU and JNU open bearing failure datasets to conduct several experiments. The experiments included small sample experiments, T-SNE visualization, confusion matrix visualization, noise perturbation experiments, and ablation experiments, and several experiments were conducted to prove the effectiveness and reliability of the method.

### 4.1. Experiments with the CWRU Dataset

#### 4.1.1. CWRU Bearing Dataset

**Figure 8:**
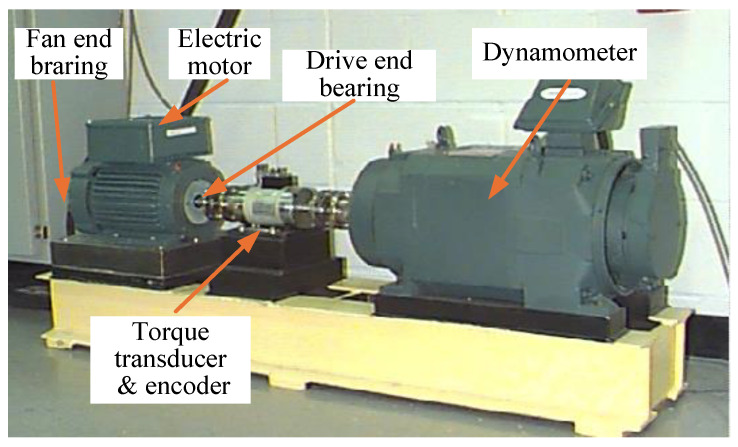
CWRU bearing test bench.

As illustrated in Figure 8 for the bearing test bench, the experimental portion of this section made use of the Case Western Reserve University (CWRU) bearing dataset [37], which is separated into 12 KHz and 48 KHz sampling frequencies at the drive end (DE) and 12 KHz sampling frequencies at the fan end (FE), as well as the bearing’s normal operation (Normal) data.

The failure point was determined using EDM etching technology on the bearing inner race (inner race, IR), outer race (outer race, OR), and rolling element (ball, Ball), with the manufacturing diameters of 0.007 inches, 0.014 inches, and 0.021 inches, respectively. This resulted in ten different types of bearing failure states. The motor horsepower of the experimental bearings was 0 HP, 1 HP, 2 HP, and 3 HP, respectively, under the four different working conditions used to collect fault signals, which correspond to motor speeds of 1797, 1772, 1750, and 1730 rpm, respectively. The outer ring fault was divided into three, six, and twelve o’clock fault positions, such that @ 6 was the outer ring 6 o’clock fault position.

For the experiment to test the model’s robustness in assessing load situations, ten different types of bearings in various fault states (under the collection of sample points) were chosen, with a 1 HP load, a speed of 1772 rpm, and the original fault signal as the working conditions. The drive end was chosen as the sampling point, with a sampling frequency of 12 KHz. Each sample had 1024 data points, and the number of samples for each class was 200, 150, 50, and 10 for the experimental samples, respectively. The sample data were then divided and are displayed in Table 2 below.

#### 4.1.2. Data Sampling and Enhancement

A multi-window sliding window overlap sampling technique is suggested as a solution to the issue of incomplete fault information collection. In addition to being sampled under numerous windows and differential overlap rates, the original data were also simultaneously normalized and spliced. The sliding step *S* and the associated number of samples per window $S_{a,i}$ were as follows, where the length of the original data is *N*, the time step is *W*, and the overlap rate is $r_{i}$.

(23) $$S_{i}=W\cdot \left(1-r_{i}\right)$$

(24) $$S_{a,i}=\left\lfloor \frac{N-W}{S_{i}}\right\rfloor +1$$

For the *i*th window, let *j* be the sample index and generate the sample $X_{i,j}$ as

(25) $$X_{i,j}=\mathrm{data}\left[j\cdot S_{i}:j\cdot S_{i}+W\right]$$

**Figure 9:**
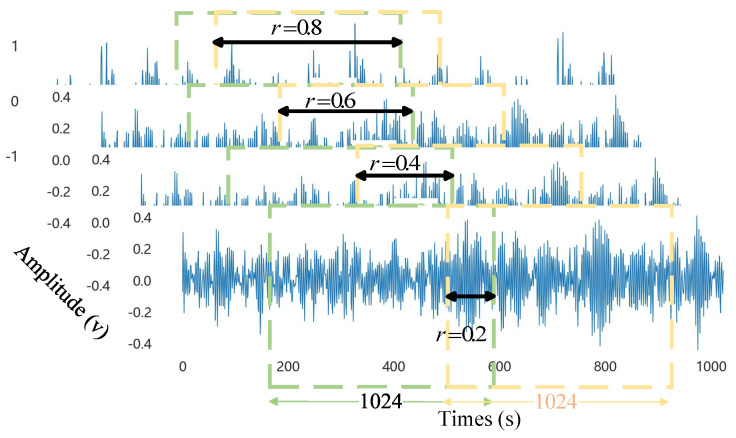
Multi−window sliding window overlapping sampling.

Since the sampling frequency of general bearing fault data is relatively high, selecting a step size of 1024 ensured that each window contained sufficient sampling points to characterize the feature correlations of the bearing vibration signal, while also matching the input dimensions of the model parameters. Setting different overlap rates enhances the richness of data at different scales and avoids the loss of key information at different nodes. Therefore, we set the multi-window time step size to 1024, with overlap rates of 0.2, 0.4, 0.6, and 0.8, respectively. The B007 scrolling body fault signal is shown as an example in Figure 9 of this paper’s multi-window sliding window overlap sampling principle.

In this case, the normalized dimension was spliced as a new fault sample, and the signal was recovered by selecting a multi-window sliding window with varying overlap rates. This approach completed the data enhancement without altering the signal’s fundamental characteristics, preserved the data source’s features as much as possible, expanded the model’s extraction range for sparse samples in the event that there were insufficient samples, and enriched the input fault features.

This study used Gaussian noise to execute enhancement operations on the read-in data, in order to avoid the challenge of feature extraction when there are insufficient fault samples and to improve the robustness against model noise:

(26) $$I_{\mathrm{noisy}}=I+N\left(0,\sigma^{2}\right)$$

where *I* is the original input signal, $N\left(0,\sigma^{2}\right)$ denotes the random noise with mean deviation 0 and variance $\sigma^{2}$, and $I_{noisy}$ is the signal after adding Gaussian noise.

#### 4.1.3. Small Sample Experiment

A Convolutional Neural Network (CNN), Convolutional Neural Net-work-Long Short-Term Memory (CNN-LSTM), Convolutional Neural Network-Gated Recurrent Unit (CNN-GRU), Bidirectional Gated Recurrent Unit-Attention (BiGRU-Attention), and the model proposed in this paper were chosen as a comparison in order to analyze the accuracy and superiority of the model in fault diagnosis tasks. The experiments were conducted with identical parameter settings, including the data preprocessing, training rounds, learning rate, optimizer selection, and batch size. As can be seen in Table 3, and the experimental accuracy radar chart, the trials were conducted under identical conditions, and the average of each set of experiments was calculated five times.

As shown, the model proposed in this paper had a test accuracy of 100% with sample numbers of 200, 100, and 50, and the average accuracy of the four groups of experiments was as high as 99.742%, which was higher than that of the other models. The accuracy of the remaining four models decreased significantly with the reduction in the number of samples, which indicates that the comparison model had difficultly learning the deep features of the fault signal during the training process, due to the scarcity of samples and the poor feature extraction ability of the model, and with a reduction in samples, the model’s ability to extract features of the bearing fault decreased sharply. However, the author’s proposed method still had a 98.966% recognition accuracy with only 10 samples, which was 14.581%, 14.281%, 20.846%, and 13.538% higher than the other models, respectively, verifying that the present model had a higher fault diagnosis accuracy with a small number of samples, and it had excellent deep-mining ability for the local and global fault features of bearings.

#### 4.1.4. T-SNE Visualization

**Figure 10:**
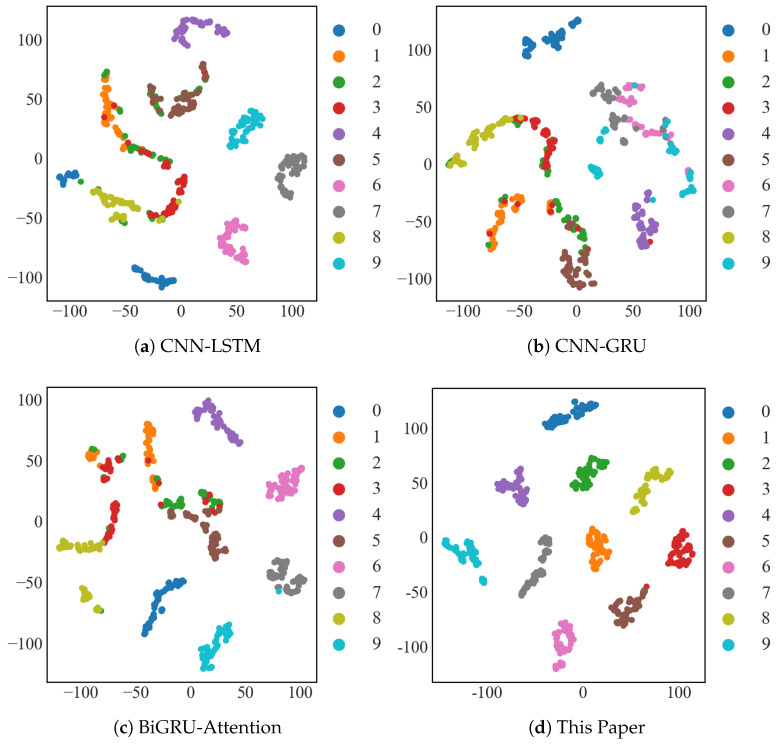
Visualization of T−SNE in the CWRU dataset.

As illustrated in Figure 10, the T-SNE (T-distributed Stochastic Neighbor Embedding) graph method [38] was used to visualize the diagnostic results of the CWRU dataset with 10 samples for CNN-LSTM, CNN-GRU, and BiGRU-Attention, as well as the T-SNE visualization graph of the model in this paper for 10 samples of the CWRU dataset. This is performed in order to visually represent the feature extraction ability of the method suggested in this paper for small-sample input signals. For the T-SNE plots, since the axes no longer have a physical meaning in relation to the original data, the scale numbers on the axes of the T-SNE plots are only provided to indicate the relative positions of the data points. Here, we mainly focus on the clustering effects of each type.

The CNN-LSTM and CNN-GRU models were not very good at classifying type 1, 2, and 3 faults, as shown in Figure 10a,b; the BiGRU-Attention model was not good at classifying type 2 and 3 faults, as shown in Figure 10d. The results show that the method of this study had a significant advantage over previous models in terms of accuracy, diagnostic effect, and perceptual learning ability for scarce bearing fault signals.

#### 4.1.5. Confusion Matrix Visualization

**Figure 11:**
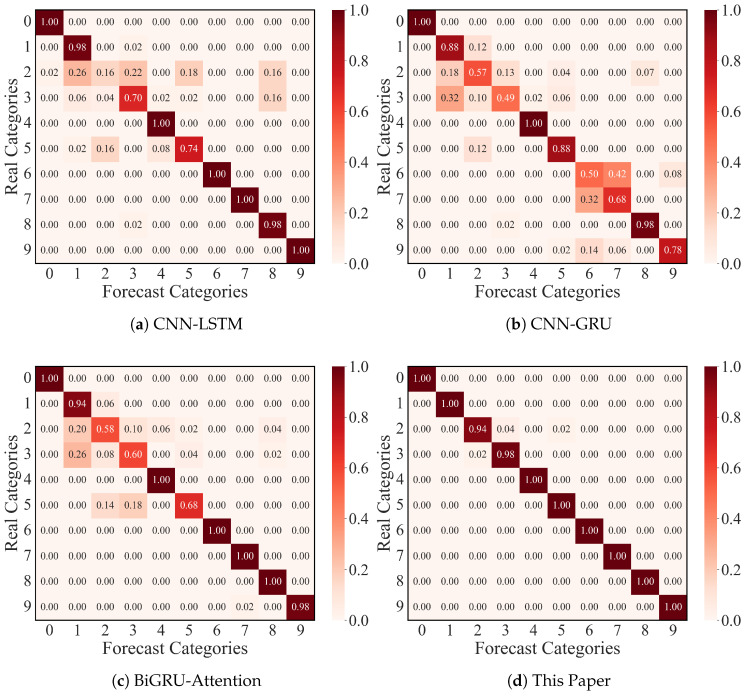
Confusion matrix for fault classification in the CWRU dataset.

To further verify the excellent classification performance of this paper’s model under a small number of samples, this paper conducted confusion matrix experiments, and Figure 11 shows the confusion matrix images of the CNN-LSTM, CNN-GRU, BiGRU-Attention, and this paper’s ResNet-CACNN-BiGRU-SDPA model for 10 samples (shown in the recognition ratio).

The CNN-LSTM model had a low recognition rate for label 2 and label 5 fault types, according to the experimental results; the CNN-GRU and BiGRU-Attention models had a low recognition accuracy for label 2, 3, and 5 types; and the ResNet-CACNN-BiGRU-SDPA model, which is suggested in this paper, was accurate in the recognition of ten classes of bearing fault types, with over 98% of the population being recognized. Based on the excellent bi-directional parallel mining ability for fault features of the dual-domain fusion model of this paper, the experiment demonstrated that the model could complete the fault diagnosis task with a small number of samples near the limit, further confirming the model’s excellent feature extraction performance.

#### 4.1.6. Noise Experiment

**Figure 12:**
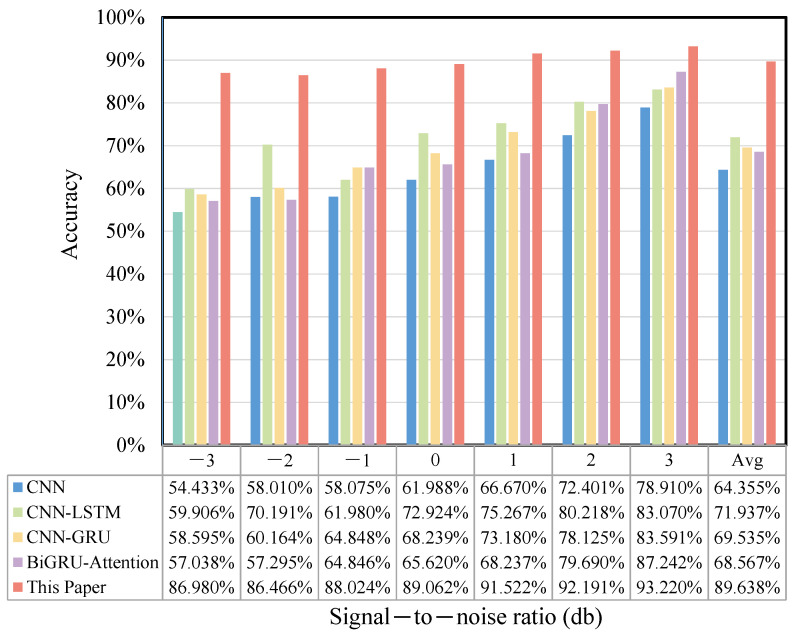
Accuracy rate for the CWRU noise experiment.

Ten types of bearing fault data under a 1 HP load 1772 rpm speed condition were used as experimental samples. This aimed to simulate environmental noise in real engineering applications and to test the robustness and accuracy of the method under various noise perturbation conditions. The comparison model and parameter settings were the same as those mentioned above, and 100 fault samples were chosen. The various signal-to-noise ratios were set to be −3, −2, −1, 0, 1, 2, and 3 db. Figure 12 illustrates the accuracy of this paper’s model, as well as the CNN, CNN-LSTM, CNN-GRU, and BiGRU-attention models under the various noise perturbations mentioned above.

From the figure, it can be seen that the accuracies of the four comparison models showed a rapidly decreasing trend when the influence of the signal-to-noise ratio gradually increased, which was greatly affected by noise. The method proposed in this paper extracted local features in parallel through the time–frequency bidirectional sensing field, and captured the dynamic weights to the spatio-temporal features through the SDPA mechanism, which maximally avoided the perturbation of the baseline features of the data by changing noise signals. In addition, the multi-window sliding-window overlapping sampling method proposed in this paper also enriched the model input signals to a greater extent, which was conducive to the preservation of the key features. The results of the ablation experiments showed that this paper’s method only had a maximum diagnostic accuracy difference of 6.240% when different signal-to-noise ratios were used, and the recognition effect was stable, which verified that this paper’s method has excellent feature characterization capability and robustness under complex noise perturbation conditions.

#### 4.1.7. Ablation Experiments

**Figure 13:**
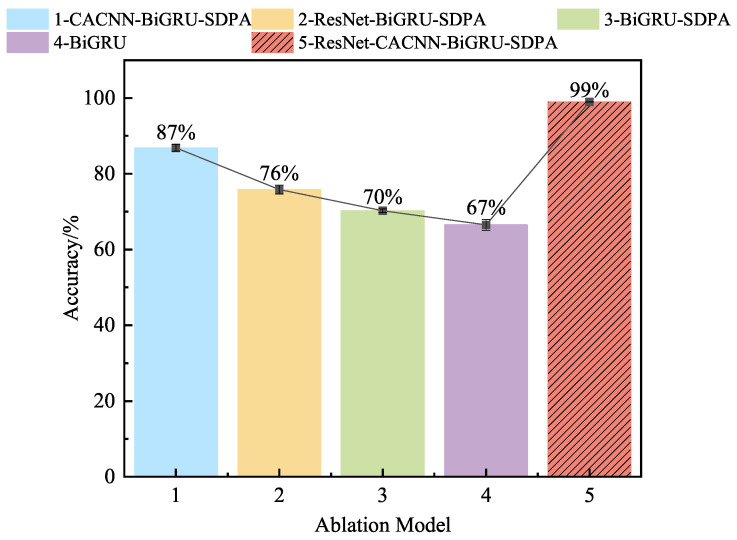
The accuracy rate for ablation experiments on the CWRU dataset.

As depicted in Figure 13 for the ablation experiment image, we continued to conduct ablation experiments with the working condition of a 1HP signal with a sample number of 10 and the CWRU dataset, in order to elucidate the degree of influence and contribution of each module in the ResNet-CACNN-BiGRU-SDPA model proposed in this paper to the fault diagnosis performance.

The images demonstrate that, in small sample conditions, the accuracy of this method was significantly better than that of the comparison models.As the experiment progresses, the core components of the model were gradually removed, and the accuracy of the model decreased significantly. It is clear that each module supports the method described in this paper and that each module is necessary for improving the overall performance of the model, thereby verifying the effectiveness of this method.

The model can maximize access to the signal features and the dependency relationship between the time and frequency domains thanks to the suggested local feature extraction method of time–frequency dual domains and feature fusion. Additionally, the SDPA mechanism’s dynamic perception enables the model to quickly update the feature weight relationship to prevent feature disappearance. The data in the figure show that when only one channel in the time or frequency domain was used to extract the features, the diagnostic accuracy rate dropped significantly. This further confirms the superiority of the current time–frequency fusion feature sensing and key feature learning method.

### 4.2. Experiments with the JNU Dataset

#### 4.2.1. JNU Bearing Dataset

**Figure 14:**
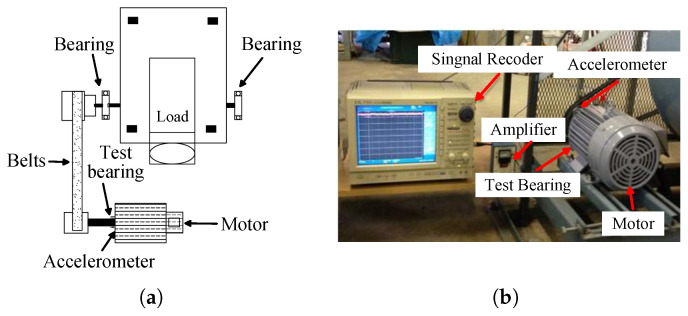
JNU dataset test bench. (**a**) Sketch of the test bench. (**b**) Sensor mounting position and motor viewpoint angle.

The Jiangnan University (JNU) bearing dataset [39], which was gathered from the failure signals of a centrifugal fan system employing a Mitsubishi SB-JR induction motor, was used in the second experiment of this work. A test rig sketch is displayed in Figure 14a, and the motor was a 3.7 kW three-phase induction motor. The entire experimental setup contained one damaged bearing, which was found in the motor’s output shaft. Figure 14b depicts the sensor mounting location and the perspective of the motor.

**Figure 15:**
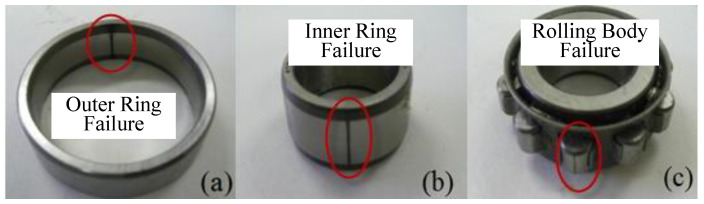
Faulty bearing. (**a**) Outer ring failure. (**b**) Inner ring failure. (**c**) Rolling body failure.

The four different fault signals of the roller bearings—normal (n), inner ring failure (ib), rolling element failure (tb), and outer ring failure (ob)—were produced by artificial wire-cutting in this experiment. The faulty bearings are displayed in Figure 15 below. The dataset was collected under three operating conditions with a sampling frequency of 50 kHz and rotational speeds of 600, 800, and 1000 rpm.

The ten experimental sample types chosen for bearing fault diagnosis in this experiment were the inner and outer ring and rolling body fault signals at 600, 800, and 1000 rpm speeds, as well as the normal bearing signals at 1000 rpm speeds. Multi-window sliding window overlapping sampling was chosen as the sampling method; the time step *W* was set to 1024; the overlap rate $r_{i}$ was 0.2, 0.4, 0.6, and 0.8, respectively; the data were normalized after slicing; 200, 100, 50, and 10 samples were chosen; the division ratio was unchanged from Experiment 1; and the sample data are displayed in Table 4.

#### 4.2.2. Small Sample Experiment

The CNN, CNN-LSTM, CNN-GRU, and BiGRU-Attention models were again selected as comparisons for the small sample experiments, in accordance with Section 4.1.3. The experiments were conducted using the same optimizer, learning rate, training rounds, and batch size parameter settings.

The ResNet-CACNN-BiGRU-SDPA model proposed in this paper also had a high recognition accuracy on the JNU dataset, with an average diagnostic accuracy of 95.812%. It also maintained its accuracy of 94.350% when only 10 samples were used, as illustrated in Table 5 for the accuracy of each model in the small sample experiment. It was experimentally confirmed that the method’s fault diagnosis and time–frequency feature mining capabilities were not significantly impacted by changes in the dataset, had good generalization, and could be used for diagnosing various bearing signals.

#### 4.2.3. T-SNE Visualization

**Figure 16:**
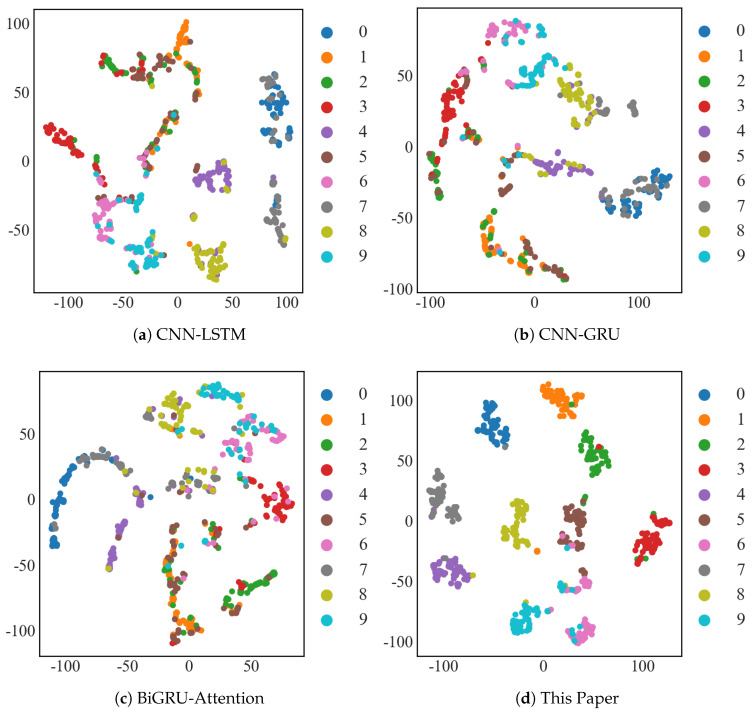
Visualization of T−SNE in the JNU dataset.

As illustrated in Figure 16, the CNN-LSTM, CNN-GRU, and BiGRU-Attention models were chosen for T-SNE visualization experiments on the JNU dataset under a 10-sample condition in this research.

It is evident from the following figure that the model used in this paper also had good fault clustering on the JNU dataset.

#### 4.2.4. Confusion Matrix Visualization

**Figure 17:**
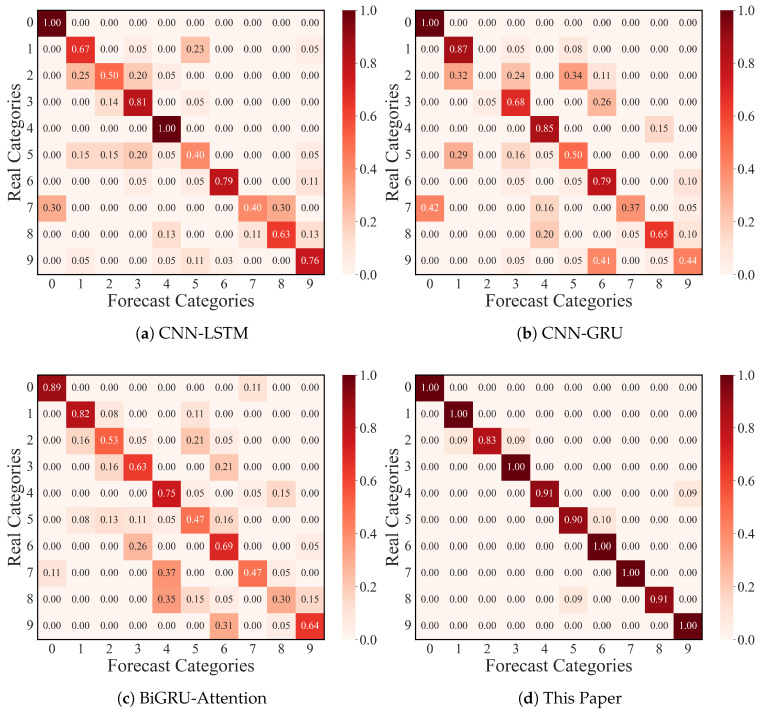
Confusion matrix for fault classification in the JNU dataset.

The confusion matrix experiment, which was based on the JNU dataset and had ten samples, is depicted in Figure 17. The CNN-LSTM, CNN-GRU, and BiGRU-Attention models in this experiment were unable to correctly identify the bearing fault types 2, 3, 7, and 8, but the current model was able to identify the ten fault types more accurately, demonstrating its superior fault diagnosis performance once more.

#### 4.2.5. Noise Experiment

**Figure 18:**
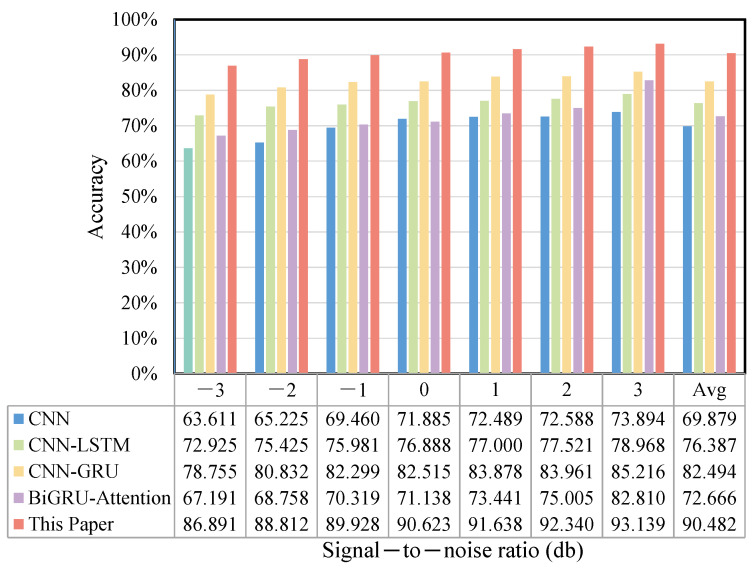
The accuracy rate of noise experiments on the JNU dataset.

The robustness and generalization ability of the model in complex environments were also tested using the noise perturbation experimental method. The model parameters remained the same, there were 100 samples, and the comparison models were CNN, CNN-LSTM, CNN-GRU, BiGRU-Attention, and this model. The diagnostic accuracies of this paper’s model and the comparative models under various noise perturbations are displayed in Figure 18, where the signal-to-noise ratio is plotted on the horizontal axis. The maximum noise difference under various signal-to-noise ratios was 6.248%, which is a good anti-noise performance, with a higher diagnostic accuracy and robustness compared with the other four models. It is evident that the diagnostic accuracy of this model of this paper was not significantly affected under different noise perturbations.

#### 4.2.6. Ablation Experiments

**Figure 19:**
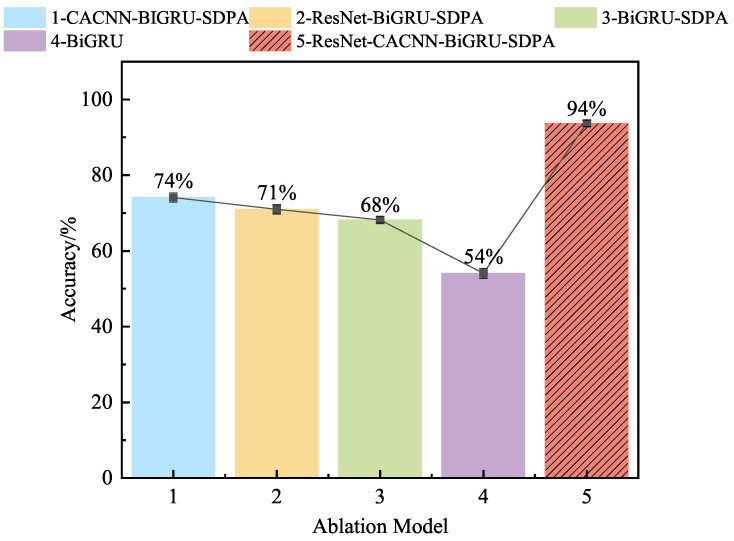
The accuracy rate of ablation experiments on the JNU dataset.

The results of the ablation experiment are shown in Figure 19. This experiment demonstrated that, similarly to the ablation experiment results on the CWRU dataset, as the core modules were progressively ablated, the contributions of each module became increasingly evident, further validating the superior performance of the proposed model and the necessity of its individual submodules. Furthermore, the proposed ResNet-CACNN-BiGRU-SDPA method, based on time–frequency dual domains and feature fusion, demonstrated a stable diagnostic accuracy when handling complex signals across various datasets. Additionally, the proposed time–frequency bidirectional mining module significantly enhanced the model’s performance, addressing issues related to fault feature extraction and diagnostic sample scarcity. Furthermore, the method also holds practical value for engineering applications.

## 5. Conclusions

This paper proposed a ResNet-CACNN-BiGRU-Attention bearing fault diagnosis method based on time–frequency dual domains and feature fusion to address the issues of sparse bearing fault samples and the high difficulty of learning fault features due to the influence of noise on a model. The authors carried out several experiments based on the CWRU and JNU bearing datasets and came to the following conclusions:

1. The multi-window sliding-window overlapping sampling method proposed in this paper enriched the original input features, and the experiments demonstrated that this method could mine more important information of the faults under the condition of a small number of samples. This paper extended the feature mining domain from the time domain to the frequency domain, giving the model a bidirectional sensing field in the time–frequency domain for signal feature extraction;
2. With the goal of maximizing the depth of signal exploration in complex environments, we analyzed the signal features in time–frequency dual domains in this paper. Then, we designed ResNet and CACNN modules to extract the local fault features in the time–frequency domain and obtained the global feature dependence of the time–frequency long sequence through a two-layer BiGRU. It was experimentally demonstrated that the model could produce better diagnostic results with greater generality and robustness across a variety of datasets under various noise perturbation settings;
3. In order to dynamically capture the spatio-temporal feature correlation weights in the dot-product state and further optimize the feature long-range dependence following BiGRU layer processing, we employed the SDPA in this paper. This helped to mitigate the perturbation of the data baseline features caused by fluctuating noise signals. The model’s strong diagnostic accuracy and noise immunity across several datasets were experimentally confirmed.

Although the above experiments proved that the method in this paper could achieve good recognition results with small samples and complex noise environments, in practical applications, the fault data acquisition sources may come from a variety of information, including temperature, pressure, images, etc., and the classification performance of the method for multiple sources of fault signals may decline and the model learning time may become longer. Thus, it is necessary to continue to optimize our research strategy and deepen the level of practical industrial applications.

In the future research, the authors will conduct further in-depth studies in the following directions: (1) This study did not investigate diagnostic efficiency in multimodal input situations, due to limitations. In future work, we will incorporate data collected from multiple sensors in industrial production to achieve fault analysis and diagnosis under multimodal input conditions, thereby improving the robustness and accuracy of the model under more complex working conditions, constructing a fault diagnosis system that is more suitable for actual industrial production. (2) We will use advanced feature extraction and enhancement techniques to further enrich the model fault features, meet the needs of fault diagnosis under further industrial scarce sample conditions, and improve the reliability of intelligent fault diagnosis; (3) We will optimize the model diagnostic logic, strive to use the simplest model to deal with complex data, and speed up the training speed of the model, while maintaining the higher accuracy of the model. (4) We will design integrated and portable modules, optimize computational resources, and improve the real-time application functionalities of models in embedded systems. (5) Our diagnostic work primarily targeted human-induced defects; however, to enhance the model’s generalization capabilities in the presence of compound or evolving faults, we will concentrate on compound fault state information in future research and broaden the context of the model’s application.

## Figures and Tables

**Table 1:** Model parameter settings.

Module	Name	Number of Network	Nuclear Size	Number of Nuclear	Output Size
Input Layer	Original Input Signal	input	-	-	1024 × 10
CACNN Layer	CNN1/CNN2	2 × Conv1d	3 × 3	16	512 × 16
	MaxPool1	MaxPool1d	2 × 2	16	256 × 16
	CNN3/CNN4	2 × Conv1d	3 × 3	32	256 × 32
	MaxPool2	MaxPool1d	2 × 2	32	128 × 32
	CNN5/CNN6	2 × Conv1d	3 × 3	64	128 × 64
	MaxPool3	MaxPool1d	2 × 2	64	64 × 64
	CoordAttention	Conv1d	1 × 1	64	64 × 64
ResNet Layer	ResidualBlock1	2 × Conv1d	3 × 3	32	1024 × 32
	ResidualBlock2	2 × Conv1d	3 × 3	64	1024 × 64
	ResidualBlock3	2 × Conv1d	3 × 3	128	1024 × 128
T-S BiGRU layer	Time-BiGRU1	BiGRU	-	128	1024 × 256
	Time-BiGRU2	BiGRU	-	64	1024 × 64
	Space-BiGRU1	BiGRU	-	128	64 × 256
	Space-BiGRU2	BiGRU	-	64	64 × 128
SDPA layer	SDPA-Time	SDPA	-	128	1024 × 128
	SDPA-Space	SDPA	-	128	64 × 128
Output layer	Fully Connected Layer	Linear	10	-	1 × 10

**Table 2:** Fault sample data of the CWRU dataset.

Fault Type	Category	Fault Size (mm)	Labeling
Normal	Normal	-	0
Rolling body failure	B007	0.1778	1
Rolling body failure	B014	0.3556	2
Rolling body failure	B007	0.5334	3
Inner ring failure	IR007	0.1778	4
Inner ring failure	IR007	0.3556	5
Inner ring failure	IR007	0.5334	6
Outer ring failure	OR007@6	0.1778	7
Outer ring failure	OR014@6	0.3556	8
Outer ring failure	OR021@6	0.5334	9

**Table 3:** The accuracy rate of small sample experiments on the CWRU dataset.

Model	200 Sample	100 Sample	50 Sample	10 Sample	Average
CNN	97.927%	93.233%	92.010%	84.385%	91.889%
CNN-LSTM	96.966%	94.792%	92.212%	84.685%	92.164%
CNN-GRU	98.701%	96.282%	93.255%	78.120%	91.590%
BiGRU-Attention	98.244%	95.832%	86.469%	85.428%	91.493%
Model of this paper	100.000%	100.000%	100.000%	98.966%	99.742%

**Table 4:** Fault sample data of the JNU dataset.

Rotation Speed	Category	Fault Size (mm)	Labeling
1000	n10002	-	0
600	ib6002	0.25	1
600	tb6002	0.15	2
600	ob6002	0.25	3
800	ib8002	0.25	4
800	tb8002	0.15	5
800	ob8002	0.25	6
1000	ib10002	0.25	7
1000	tb10002	0.15	8
1000	ob10002	0.25	9

**Table 5:** The accuracy rate of small sample experiments on the JNU dataset.

Model	200 Sample	100 Sample	50 Sample	10 Sample	Average
CNN	82.186%	77.706%	69.376%	58.970%	72.060%
CNN-LSTM	84.062%	81.874%	62.500%	61.390%	72.457%
CNN-GRU	89.060%	76.664%	63.124%	66.410%	73.815%
BiGRU-Attention	86.252%	84.900%	79.372%	64.520%	78.761%
Model of this paper	97.866%	96.040%	94.990%	94.350%	95.812%

## Data Availability

The CWRU Bearing Dataset is available for open access at https://engineering.case.edu/bearingdatacenter (accessed on 15 April 2025). The JNU Bearing Dataset is available for open access at https://github.com/Tan-Qiyu/Mechanical_Fault_Diagnosis_Dataset (accessed on 15 April 2025).
